# Compartment and Crush Syndromes After Sleep Deprivation and a Therapeutic Dose of Zolpidem

**DOI:** 10.5811/cpcem.2017.4.30837

**Published:** 2017-07-06

**Authors:** Martin R. Huecker, Eric Yazel

**Affiliations:** University of Louisville School of Medicine, Department of Emergency Medicine, Louisville, Kentucky

## Abstract

Despite extensive review in the literature, compartment syndrome and crush syndrome remain difficult to diagnose. Trauma, toxins and reperfusion have been associated with these syndromes. Cases involving alcohol and drug abuse have described patients “found down” compressing an extremity. We present a case of a registered nurse who developed compartment syndrome in multiple limbs due to prolonged sleep after sleep deprivation and zolpidem use. To our knowledge, this is the first case of compartment syndrome or crush syndrome to have occurred in the setting of zolpidem use. Sleep disruption in healthcare workers represents a public health issue with dangerous sequelae, both acute and chronic.

## INTRODUCTION

A shift worker is defined as “anyone who works extended-duration shifts and other variable and nonstandard hours … late into the night or very early in the morning.”^1^ Nurses and hospital staff implemented shift-based scheduling long ago. Emergency physicians (EP) adopted the practice early in the evolution of the specialty. Hospitals are non-stop businesses with high rates of error occurring at very high stakes. Hospital staff and physicians who work night shifts and swing shifts are subjected to circadian disruption that leads to fatigue, poor performance, and patient harm. Many shift workers find themselves unable to obtain satisfactory quality or quantity of sleep due to rotating schedules. Sleep deprivation has been shown to impair vigilance, cognition, memory and fine motor skills.^2^ Night shift work and subsequent circadian disturbance have independent deleterious effects on these parameters.^2^ Cognitive performance decline from sleep deprivation appears similar to alcohol intoxication, with 17 hours of wakefulness correlating to a blood alcohol concentration of 0.05% and 24 hours to 0.10%.^3^

Compartment syndrome refers to the pressure increase in a closed fascial space to the point of reduced capillary perfusion.^4^ Usually due to long bone fracture, these pressure increases can ensue when a limb is crushed by a person’s own bodyweight. With severe or prolonged crushing force, muscle necrosis occurs with subsequent life-threatening systemic effects: rhabdomyolysis, renal failure, hyperkalemia, and death. We present the case of a registered nurse who used pharmaceuticals to “catch up” on sleep, waking up 30 hours later with crush and compartment syndromes in multiple limbs.

## CASE REPORT

An African-American female in her early thirties with normal body mass index and no past medical history sought rest after 30 hours without sleep. The patient took 50 milligrams (mg) of diphenhydramine and five mg of immediate-release zolpidem. She awoke unable to move her right upper and lower extremities and had severe pain in the right side of her body. The patient contacted staff in her emergency department (ED) and was encouraged to call emergency medical services for transport to the hospital.

Initial vital signs were as follows: temperature 98.0 F, heart rate 112 beats/minute, respirations 14/minute, blood pressure 130/76 mmHg, oxygen saturation 96% on room air. Physical exam was remarkable for tense swelling over the right chest, shoulder and proximal arm. The induration extended to her right flank and proximal gluteal area. The patient was unable to lift her right upper and lower extremities against gravity. Minimal digit flexion/extension was achievable. Radial and ulnar pulses were not palpable but could be obtained with Doppler.

Initial laboratory findings were significant for leukocytosis (45.2 × 10^3^ /mcL), potassium 6.1 mEq/L, bicarbonate 18 mmol/L, blood urea nitrogen (BUN) and creatinine (Cr) = 47mg/dL and 4.3 mg/dL. Urinalysis showed large blood and >50 red blood cells per high power field; lactate was 4.5 mmol/L and total creatine kinase (CK) was 346,866 IU/L. Aspartate aminotransferase and alanine aminotransferase were 3,200 U/L and 1,145 U/L. The EP was concerned for both a cerebrovascular event and compartment syndrome. Due to objective hemiparesis and rapid availability of magnetic resonance imaging (MRI), MRI brain, cervical spine, and right shoulder were obtained. MRI brain and spinal canal were normal, but the soft tissues and muscles of the neck and the shoulder displayed extensive edema concerning for myonecrosis.

Central venous access was obtained and the patient received normal saline with sodium bicarbonate. A urinary catheter yielded 50cc of tea-colored urine. The treatment team initiated transfer to a tertiary care center for hand surgery consultation and fasciotomy, nephrology evaluation, and intensive care unit coordination of care.

On arrival to our academic center, right upper extremity and right lower extremity compartment pressures were measured with a Stryker Intra-Compartmental Pressure Monitor: 75 and 35mmHg, respectively. Surgical consultants performed fasciotomies of the following compartments: right lateral thigh, right upper arm anterior and posterior compartment, right forearm volar and dorsal, and right hand thenar, hypothenar, dorsal, and volar interosseous compartments. Bicarbonate-rich intravenous (IV) fluids were continued. By hospital day three, her BUN and Cr had increased to 72 mg/dL and 7.5 mg/dL; total CK was 115,000 IU/L. Her urine output decreased.

The patient gradually regained sensation and motor function of the right upper and right lower extremities. She underwent multiple debridements and eventual closure of her fasciotomy sites. Her course was complicated by multiple episodes of symptomatic anemia requiring transfusion. She was discharged on hospital day 18. The last total CK was checked on hospital day eight and was 6,848 IU/L. Discharge BUN was 15 mg/dL and Cr was 1.3 mg/dL. One month after the injury the patient could ambulate with minimal limp and write and perform other fine motor tasks at near preinjury levels.

CPC-EM Capsule

What do we already know about this clinical entity?

Compartment and crush syndromes can be difficult to diagnose. In addition, sleep deprivation and circadian disruption affect all emergency care providers.

What makes this presentation of disease reportable?

To our knowledge, this is the first case of compartment and crush syndromes to have occurred in the setting of sleep deprivation or zolpidem use.

What is the major learning point?

Sleep disruption in healthcare workers represents a public health issue with dangerous, potentially fatal sequelae, both acute and chronic.

How might this improve emergency medicine practice?

EM practitioners should have suspicion for these syndromes. In addition, we should protect ourselves against the consequences of shift work.

## DISCUSSION

To our knowledge, this is the first case of compartment or crush syndrome associated with the use of zolpidem. Compartment syndrome has been described in the setting of diphenhydramine overdose with coingestion of alcohol.^5^ We believe zolpidem and diphenhydramine had synergistic effects in this case that led to prolonged down time, although we cannot conclude a direct causal link between zolpidem and rhabdomyolysis. This case is also remarkable for the presence of compartment syndrome in multiple extremities induced only by pressure from body weight. Cases of multiple-limb compartment syndrome typically occur in natural disasters and collapsed buildings, or in patients with preexisting hematologic disease.^6^,^7^ The sadly *common* aspect of our case is the sleep deprivation and circadian disruption that led to our patient’s presentation. Sleep loss causes many acute performance and health effects. Memory and learning are disrupted, performance and judgment are impaired, and musculoskeletal injuries are more common.^8^ The cycle of poor sleep and shift work leads to chronic health consequences: obesity, metabolic syndrome, cardiovascular disease, gastrointestinal disease, dysmenorrhea, psychological disorders, cancer, and motor vehicle collisions.^1^,^8^,^9^ Chronic low levels of sleep loss cause similar impairments to acute sleep loss, and synergy exists between acute sleep loss and chronic sleep loss with circadian disruption.^8^ Two weeks of sleeping less than six hours daily equates to the performance deficit of 24 continuous hours without sleep.^10^ One week of sleeping four hours per night yields the performance deficit similar to 48 consecutive hours without sleep.^10^ Daytime sleep following a night shift is typically of short duration (5.5–6 hours) and low quality.^11^ This contrasts with what occurred in the case presented.

The impaired clinical performance in sleep-deprived healthcare workers impacts patient safety. Errors by resident physicians are seven times more common in those working five or more 24-hour shifts in a month.^9^ Needlesticks and motor vehicle accidents also increase with fatigue.^9^ A review by Lockley et al. collated the many negative effects of working recurrent 24-hour shifts: five times the diagnostic errors, twice the attentional failures, 61% more needlestick injuries, and 300% more fatigue-related preventable adverse events leading to patient demise.^10^ Fatigue is underappreciated in the person who is fatigued. Study participants in the lowest alertness category often rate themselves as “only slightly sleepy.”^8^ In 2011, the Joint Commission issued a *Sentinel Event Alert* to draw awareness to shift length and work schedule effect on quality and quantity of sleep.^12^ Nurses working 12-hour shifts have higher rates of burnout and job dissatisfaction.^13^ In the *Sentinel Event Alert*, the Joint Commission urged healthcare organizations to educate staff on sleep hygiene and how fatigue impacts patient safety.^12^ A fatigue management plan should be followed and fatigue should be investigated as a contributing factor in review of all adverse events.^12^

Pharmaceuticals are used in an attempt to mitigate fatigue and sleep disruption. Stimulants to increase alertness are cycled with sedatives to aid with sleep, often to the detriment of the person using the substances. In one survey of 226 emergency medicine residents, the use of sedatives approached 36%.^14^ The American Academy of Sleep Medicine (AASM) cautions against the use of sedative-hypnotics due to side effects.^15^ Sedative-hypnotics can worsen sleep-related breathing issues or cause subjective mood worsening, and do not reliably improve performance and safety.^15^ Zolpidem is a sedative-hypnotic drug, FDA approved since 1992 for short-term treatment of insomnia.^16^ Side effects range from daytime drowsiness, dizziness, hallucinations, agitation, and sleepwalking to committing crimes and driving or eating while asleep.^16^ To our knowledge, zolpidem has not been implicated in a case of compartment or crush syndrome. Though some medications have been implicated in direct muscle damage, we believe zolpidem in this case caused a sedative effect and prolonged downtime. Considering all of the evidence for and against zolpidem use, the AASM includes zolpidem in its collection of drugs with minimal benefit in shift-work disorder.

### Compartment Syndrome

From Volkmann describing the contracture in 1881 to the current day, compartment syndrome has been challenging to diagnose.^17^,^18^ The morbidity of a nonviable limb along with potential for death make compartment syndrome a prominent medico-legal concern, with an average payout of $426,000.^18^ Compartment syndrome most often occurs after an extremity injury, though 30% of cases have no evidence of fracture.^17^ Less common causes include infection, surgical positioning, constricting casts or splints, bleeding diathesis, reperfusion, toxins and burns.^17^,^19^ The classic “P’s” (pain, paresthesia, pallor, paresis and pulse deficit) are not adequately sensitive and are often found only after irreversible muscle damage has occurred.^17^ The patient will often have pain at rest and out of proportion to the physical examination findings. Passive stretching of the muscles in the involved compartment is one of the earlier signs.^17^ The consensus on diagnosis of compartment syndrome designates clinical exam as the most important factor. Measurement of compartment pressures should be done in cases with equivocal exam findings, communication barriers (including pediatric patients), analgesia or anesthesia, multiple injuries, or comatose state.^4^,^17^

The decision to perform fasciotomy is made by surgical consultants when available. Most experts agree on surgical management by fasciotomy for the following cases: hypotensive patients with compartment pressure greater than 20mmHg; unconscious patients with compartment pressure greater than 30mmHg; and normotensive awake patients with positive clinical findings.^18^ All affected compartments should be decompressed.^4^ Cases of compartment syndrome in multiple extremities, as in our patient, have been described. However, this occurs almost exclusively in whole-body crush injury or in patients with predisposing comorbidities: sickle cell disease, capillary leak syndrome, and chronic myeloid leukemia.^7^ Cases of compartment syndrome in multiple extremities not associated with one of these scenarios are rare to nonexistent.

### Crush Syndrome

First described in 1941 after the Battle of London, crush syndrome occurs when local tissue damage leads to systemic effects: rhabdomyolysis, hyperkalemia, and renal failure.^6^,^19^,^20^ Commonly encountered causes are natural disasters and building collapse, or conditions causing prolonged down time: stroke, mental illness, intoxication with alcohol, heroin, or other sedatives.^6^,^19^

The manifestations of crush syndrome can be categorized by the body systems affected. Elevated serum potassium, urate and phosphorous can precipitate arrhythmias even in the first hour.^6^,^20^ The local effects of the crush injury may cause hemorrhage and third spacing, exacerbating the shock state and disrupting cardiovascular stability.^6^,^20^ Renal injury represents the complication with the highest mortality. Pulmonary, hematologic and infectious concerns also complicate the course of the crush victim. Acute respiratory distress syndrome, fat emboli, disseminated intravascular coagulation, shock liver, and wound infections can lead to morbidity and mortality in this population.^6^ After the ABCs, the crush syndrome patient needs early aggressive IV fluids.^20^ Patients may require 6–12 liters per day of IV fluids to yield a recommended urine output of 200–300 cc/hr.^6^,^7^,^20^

Treatment with mannitol and sodium bicarbonate fluids is controversial, although physiologically sound. In most studies these two interventions are combined, making it difficult to parse the role of each. No randomized controlled trial has been conducted to establish or disconfirm a benefit with either of these therapies.^6^ Both therapies remain reasonable depending on individual patient characteristics.

When hyperkalemia, volume overload or severe acidosis are present, renal replacement may be necessary, although hemodialysis does not actually remove the very large myoglobin molecule from the circulation.^6^ Our patient received sustained low-efficiency dialysis for 72 hours to treat hyperkalemia and volume overload.

Crush syndrome has a relatively good prognosis with early recognition and initiation of IV fluids, proper supportive care, and local management of compartment syndrome when present.^6^

## CONCLUSION

Acute and chronic deleterious health effects will invariably follow the sleep and circadian disruption associated with shift work. Sedative-hypnotic drugs have limited application in the maintenance of healthy sleep schedules in those working extended hours and night shifts. This case of compartment syndrome and crush syndrome represents a rare but representative example of the acute effects of sleep deprivation on the healthcare worker. Early diagnosis and aggressive management of these complications allowed for an almost complete recovery. The emergency physician must remain vigilant in considering these limb and life-threatening diagnoses, and in ensuring his/her own wellness.
